# Unsupervised high-intensity interval training improves glycaemic control but not cardiovascular autonomic function in type 2 diabetes patients: A randomised controlled trial

**DOI:** 10.1177/1479164118816223

**Published:** 2018-12-12

**Authors:** Sophie Cassidy, Vivek Vaidya, David Houghton, Pawel Zalewski, Jelena P Seferovic, Kate Hallsworth, Guy A MacGowan, Michael I Trenell, Djordje G Jakovljevic

**Affiliations:** 1 Institute of Cellular Medicine, Newcastle University, Newcastle upon Tyne, UK; 2 Department of Epidemiology, Faculty of Medicine, Nicolaus Copernicus University, Torun, Poland; 3 Cardiovascular Division, Brigham and Women’s Hospital, Harvard Medical School, Boston, MA, USA; 4 Clinic for Endocrinology, Diabetes and Metabolic disorders, Clinical Center Serbia and Faculty of Medicine, University of Belgrade, Belgrade, Serbia; 5 NIHR Innovation Observatory, Newcastle University, Newcastle Upon Tyne, UK

**Keywords:** Exercise, type 2 diabetes mellitus, cardiovascular diseases

## Abstract

**Background:**

This is the first randomised controlled trial to assess the impact of unsupervised high-intensity interval training on cardiovascular autonomic function in adults with type 2 diabetes.

**Methods:**

A total of 22 individuals with type 2 diabetes (age 60 ± 2 years, 17 males) lay in a supine position for 20 min for evaluation of cardiovascular autonomic function, which included (1) time domain measures of heart rate variability, (2) frequency domain measures of heart rate variability and blood pressure variability and (3) baroreflex receptor sensitivity. Participants were randomised into 12 weeks of high-intensity interval training (3 sessions/week) or standard care control group.

**Results:**

After 12 weeks, the between-group change in HbA_1c_ (%) was significant (high-intensity interval training: 7.13 ± 0.31 to 6.87 ± 0.29 vs Control: 7.18 ± 0.17 to 7.36 ± 0.21, *p* = 0.03). There were no significant changes in measures of heart rate variability; R-R interval (ms) (high-intensity interval training: 954 ± 49 to 973 ± 53 vs Control: 920 ± 6 to 930 ± 32, *p* = 0.672), low frequency/high frequency (high-intensity interval training: 0.90 ± 0.21 to 0.73 ± 0.07 vs Control: 1.20 ± 0.29 to 1.00 ± 0.17, *p* = 0.203), or blood pressure variability; systolic blood pressure low frequency/high frequency (high-intensity interval training: 0.86 ± 0.21 to 0.73 ± 0.10 vs Control: 1.06 ± 0.26 to 0.91 ± 0.14, *p* = 0.169). At baseline, HbA_1c_ was negatively correlated with baroreflex receptor sensitivity (*r* = –0.592, *p* < 0.01).

**Conclusion:**

High-intensity interval training improves glycaemic control but has limited effect on cardiovascular autonomic regulation in patients with type 2 diabetes.

## Introduction

Cardiovascular disease is the leading cause of death in adults with type 2 diabetes,^1^ and cardiovascular autonomic neuropathy has been shown to be a strong predictor of cardiovascular disease and mortality in type 2 diabetes.^2^ The cardiovascular autonomic nervous system has two major components: the parasympathetic and the sympathetic nervous system which control heart rate, myocardial contractility, cardiac electrophysiology, haemodynamics, and the constriction and dilation of blood vessels. Cardiovascular autonomic neuropathy occurs due to impairment of autonomic nerve fibres which innervate the heart and blood vessels, leading to abnormalities in heart rate control and vascular dynamics.^2^

Measures of heart rate variability (HRV) and blood pressure variability (BPV) allow determination of sympathovagal balance.^2^ Low frequency (LF) (0.04–<0.15 Hz) HRV and BPV reflect a balance between sympathetic and parasympathetic activity, whereas high frequency (HF) (0.15–0.40 Hz) is solely mediated by parasympathetic activity. Both HRV and BPV have been shown to be significant predictors of cardiac morbidity^3^ and cardiovascular risk.^4^ Lower HRV is associated with the development of coronary heart disease and increased rate of cardiovascular events in individuals with diabetes.^5^

Lifestyle modifications, that is, exercise and diet are the first-line treatment for patients with type 2 diabetes.^6^ Exercise has been suggested to improve cardiovascular autonomic function in healthy individuals and type 2 diabetes patients^7,8^ with the majority of studies showing improved vagal tone, demonstrated by a reduction in resting heart rate and improvement in HRV.^7,9^ It should be noted that most studies evaluating the impact of exercise in type 2 diabetes have used moderate-intensity continuous training, which is reflected in the current guidelines.^10^ More recently, high-intensity interval training (HIIT) has been shown to be a safe and effective exercise strategy to induce cardiovascular and metabolic benefits in type 2 diabetes.^11^ HIIT refers to brief intervals of vigorous activity interspersed with periods of low activity or rest and evokes a large stimulus to the heart.^12^ To date, there has been no study evaluating the impact of unsupervised HIIT on cardiovascular autonomic function in patients with type 2 diabetes. Therefore, this study was designed to assess the effect of HIIT on glycaemic control and whether this was associated with improvements in cardiovascular autonomic function.

## Methods

This was a randomised controlled trial (RCT) investigating the efficacy of HIIT in type 2 diabetes. The study was approved by the Newcastle and Northeast Tyneside Research Ethics Committee, patients provided written informed consent and all procedures were in accordance with Declaration of Helsinki (Trial registration: www.isrctn.com 78698481).

### Participant characteristics

**Figure 1.**
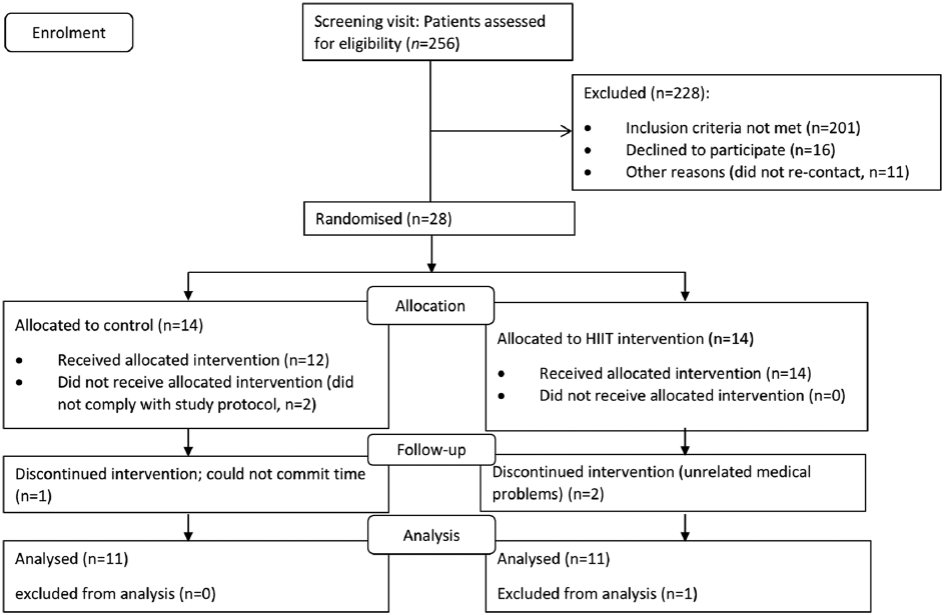
Consort flow diagram showing patient enrolment.

A total of 256 individuals with type 2 diabetes (stable control with diet and/or metformin for at least 6 months) were screened for eligibility through local adverts in newspapers and diabetes community groups, between September 2012 and September 2013. Exclusion criteria included the presence of overt cardiac disease, ⩾60 min moderate-vigorous activity per week, β-blocker therapy or contraindications to exercise stress testing according to guidelines set by the American College of Sports Medicine.^13^ Following screening, participants were randomised into the intervention (*n* = 14) or control group (*n* = 14). Six participants were lost during the study; leaving *n* = 11 in the intervention group and *n* = 11 in the control group (Figure 1). Participant baseline characteristics are listed in Table 1.

**Table 1.** Patient characteristics at baseline.

Characteristic	Control	Intervention	*p*-value
*N* (male:female)	11 (8:3)	11 (9:2)	0.611
Age (years)	59 ± 3	60 ± 3	0.87
Time since diagnosis of type 2 diabetes (years)	5 ± 1	4 ± 1	0.28
Height (cm)	169 ± 3	171 ± 2	0.77
Weight (kg)	90.8 ± 2.9	90.5 ± 4.9	0.96
BMI (kg/m^2^)	32.0 ± 1.65	31.2 ± 1.70	0.75
Body surface area	2.01 ± 0.03	2.02 ± 0.05	0.94
HbA_1c_ (%)	7.18 ± 0.17	7.13 ± 0.31	0.88
HbA_1c_ (mmol/mol)	55.0 ± 1.8	54.4 ± 3.3	0.88
VO_2peak_ (mL/min/kg)	20.3 ± 1.84	21.7 ± 1.74	0.57
Medication use			
Metformin	7	7	1.00
Statins	6	7	0.67
Anti-hypertensives	5	3	0.375

BMI: body mass index; HbA_1c_: glycated haemoglobin; VO_2peak_: peak oxygen uptake.

### Experimental protocol and randomisation

All participants were initially screened to check eligibility for the study and for any underlying cardiac disease. Participants were randomly allocated into the intervention and control groups using a random allocation sequence (www.randomisation.com) which was performed by a member of the research team who was not responsible for patient enrolment. Following the screening visit, cardiovascular autonomic function and HbA_1c_ were measured at baseline and after 12 weeks of HIIT (intervention group) or continued standard care (control group). HbA_1c_ was measured within 48–72 h of the final HIIT session. Those randomised to standard care were asked to maintain their normal routine and not change their medication, physical activity, diet or body weight. All measurements were performed at the Clinical Research Facility, Royal Victoria Infirmary, Newcastle Upon Tyne.

### Screening

Screening included evaluation of underlying cardiac disease using medical history, physical examination and a 12-lead ECG (Custo med, Ottobrunn, Germany) at rest and during exercise. Prior to exercise testing, participants completed a 20-min resting period in a supine position, where beat-to-beat blood pressure was measured by the vascular unloading technique.^14^

Maximal graded cardiopulmonary exercise stress testing was performed on a semi-recumbent cycle ergometer (Corival Lode, Groningen, Netherlands) with workload increasing by 10 W/min. Gas exchange and ventilatory data were assessed at rest and during exercise testing (Cortex metalyser 3B, Leipzig, Germany). Peak O_2_ consumption was defined as maximal oxygen consumption attained at peak exercise and averaged over the last 30 s of exercise test. The Borg scale^6–20^ was used to estimate rate of perceived exertion.^15^ The exercise was terminated when a participant reached (1) maximal oxygen consumption, (2) exhaustion participants could not maintain a cadence of 60 revolutions per min and (3) continuing further exercise was considered to be contraindicated, that is, high blood pressure response or changes on electrocardiogram suggestive of ischaemia or abnormal heart rhythm.^13^

### Cardiovascular autonomic function assessment

Cardiovascular autonomic function was measured using a Task Force® Monitor (Task Force Monitor, CNSystems Medizintechnik GmbH, Graz, Austria) which has been shown to be a reliable non-invasive method for assessing haemodynamics and autonomic function.^16^ Recordings lasted for 20 min with participants in a resting supine position.

Task Force Monitor measures beat-to-beat heart rate using a six-channel electrocardiogram, beat-to-beat and oscillometric blood pressure (to cross-check the values from beat-to-beat blood pressure measurements) using a vascular unloading method. The beat-to-beat values are then used by the Task Force Monitor to provide a power spectral analysis for HRV and blood BPV, allowing analyses of differences in the magnitude and type of variations in heart rate and blood pressure. Spectral analysis of BPV is also a reliable tool to assess sympathetic and parasympathetic regulation.^17^

HRV is analysed using time-domain and power spectral analyses. Time-domain analysis of HRV calculates the beat-to-beat interval [R-R interval (RRI) and standard deviation of the R-R interval (SDNN)]. The power spectral analysis of HRV looks at HF and LF variations in the RRI, and so are represented by RRI LF and RRI HF. Increases in the LF component are considered to be a consequence of both sympathetic and parasympathetic activities (i.e. raised during hypotension/physical activity), whereas the HF component is solely mediated by parasympathetic activity.^18,19^ The ratio between these two components, LF/HF ratio, can be used as an indicator of sympathovagal balance; a lower ratio being desirable as this represents a greater parasympathetic relative to sympathetic stimulation.

BPV is analysed using power spectral analyses for both systolic blood pressure (SBP) and diastolic blood pressure (DBP). Similar to HRV, the LF and HF variations and their ratio, LF/HF ratio, are examined. These were represented in both SBP (i.e. SBP LF, HF, LF/HF) and DBP (DBP LF, HF, LF/HF).

Baroreflex receptor sensitivity (BRS) was assessed using the sequence technique integrated into the Task Force Monitor. The sequence technique evaluates the relationship between beat-to-beat changes in BP (mmHg) and the change in RRI (ms). This relationship represents the BRS represented by ‘total slope mean’.^20,21^ A low value for total slope mean (a flatter slope) represents impairments to BRS. A high value for total slope mean (a steeper slope) is caused by an effective vagal baroreflex. This method has been shown to yield highly reproducible results compared to ‘invasive methods’ that require a phenylephrine bolus.^20,22^

### Glycaemic control

HbA_1c_ levels were measured using fasting plasma samples by a TOSOH HLC-723G8 (Minato, Tokyo, Japan). This analysis was done in an accredited clinical pathology laboratory (Department of Clinical Biochemistry, The Newcastle-upon-Tyne Hospitals NHS Foundation Trust).

### Exercise training protocol

Participants randomised to the HIIT group were required to undertake 36 cycle sessions over 12 weeks (3 sessions/week on non-consecutive days) at a local gym. Participants had to perform at least 32 sessions for inclusion in the analysis. Apart from the HIIT sessions, participants were instructed to continue their normal routine for 12 weeks, with no changes to medication, usual physical activity or diet. Adherence to sessions was monitored using an exercise diary and weekly phone calls. During exercise, the intensity was rated using the Borg scale.^15^

Each exercise session started with a 5-min warm up where the rating of perceived exertion (RPE) increased from 9 (‘very light’) to 13 (‘somewhat hard’) at a comfortable cadence, followed by five intervals where the pedal cadence was required to exceed 80 revolutions per min, and the RPE reached 16–17 (‘very hard’). Each working interval was interspersed with a 3-min recovery period consisting of 90 s of passive recovery, 60 s of band-resisted upper body exercise and 30 s of preparation for the following working interval. The band-resisted upper body exercise was very light and designed to keep participants moving during the recovery period, rather than resistance exercise per se. A 3-min recovery cycle followed the final interval. The length of the high-intensity intervals in the first week was 2 min, increasing by 10 s each week, so that by week 12 participants were performing intervals lasting 3 min 50 s.

The band-resisted (Bodymax Fitness, Clydebank, UK) upper body exercise consisted of face pull, horizontal push, horizontal pull and 30° push. One exercise was performed per recovery period in the order stated. The first session was supervised, while for the following sessions participants used voice recorded instructions available through an iPod (Apple, California, USA) to guide their sessions.

### Statistical analyses

We selected a sample size of 12 to provide a statistical power of 80% to detect a difference of 0.6% in HbA_1c_, which was our primary outcome.^23^ A sample size of 14 was used to allow for two dropouts per group.

The data from the Task Force Monitor for HRV, BPV and BRS were exported to a Microsoft Excel (Microsoft, Washington, USA) spreadsheet created for each participant. Data were screened for univariate and multivariate outliers. The outliers were defined using Tukey’s method. This was achieved using the quartile function to calculate the 1st (Q1) and 3rd quartiles (Q3), and interquartile range (IQR); outliers were identified as ‘Q1 – 1.5 (IQR) > outliers > Q3 + 1.5 (IQR)’ and subsequently deleted. The mean values of the measures of autonomic regulation and haemodynamics were then collated into a single database on an Excel spreadsheet together with other clinical characteristics before and after 12 weeks.

Statistical analyses were performed using the SPSS software (version 21, NY, USA). Data were screened for normality using the Shapiro–Wilk test. For analysing the relationship between the measures of cardiovascular autonomic function to glycaemic control, Pearson’s correlation coefficient (*r*) was used for normally distributed variables, and Spearman’s Rho (*r_s_*) was used as a non-parametric alternative when variables were not normally distributed.

The change from baseline to post-treatment was assessed for normality using the Shapiro–Wilk test. A paired Student’s *t*-test was used to evaluate differences between pre- and post-intervention for variables that were normally distributed, whereas variables not normally distributed were assessed using the non-parametric alternative, Wilcoxon signed-rank test. One-way analyses of covariance (ANCOVAs) were used to assess between-group differences which corrects for any potential differences identified at baseline comparison.^24^ *p* values < 0.05 were considered statistically significant.

## Results

### Baseline characteristics of participants

The mean age of participants was 60 ± 3 and 59 ± 3 years, with duration of type 2 diabetes of 4 ± 1 and 5 ± 1 years in the HIIT and control group, respectively. Participants in both groups were obese, body mass index (BMI) 31.2 ± 1.70 in the HIIT and 32.0 ± 1.65 in the control group. There were no significant differences in baseline characteristics between the HIIT and control cohort (Table 1). Adherence to HIIT was high, participants attained maximal attendance (36 ± 0.9 sessions) during the 12 weeks, and none dropped out.

### Effect of HIIT on body weight, glycaemic control and haemodynamic measures

Glycaemic control improved following HIIT, with a reduction in HbA_1c_ of 2.8 mmol/mol (–0.26%), compared to a 2 mmol/mol (0.18%) increase in the control group, showing a significant between-group difference (*p* = 0.03; Table 2). There were no differences in body weight or BMI change between the two groups (Table 2). Also, no difference in the haemodynamic measures (including cardiac output, heart rate, stroke volume) between groups (Table 2) was observed.

**Table 2.** The effect of HIIT on anthropometric, clinical and haemodynamic measures.

	Control (*n* = 11)				Intervention (*n* = 11)				Between-group *p*-value
	Baseline	Post-treatment	*p*-value	Δ (%)	Baseline	Post-treatment	*p*-value	Δ (%)	
Weight (kg)	90.8 ± 2.9	90.8 ± 2.8	1.00	0	90.5 ± 4.9	89.6 ± 4.8	0.042*	0.1	0.123
BMI (kg/m^2^)	32.0 ± 1.7	32.0 ± 1.7	0.929	0	31.2 ± 1.7	31.0 ± 1.7	0.125	0.6	0.216
HbA_1c_ (%)	7.18 ± 0.17	7.36 ± 0.21	0.074	2.5	7.13 ± 0.31	6.87 ± 0.29	0.151	3.6	0.03*
HbA_1c_ (mmol/mol)	55.0 ± 1.8	57.0 ± 2.3	0.074	3.6	54.4 ± 3.3	51.6 ± 3.2	0.151	5.1	0.03*
HR (beats/min)	66 ± 2.4	66 ± 2.0	0.482	0	65 ± 4	64 ± 4	0.556	1.5	0.841
SBP (mmHg)	119 ± 5	122 ± 4	0.52	2.5	118 ± 5	119 ± 4	0.748	0.8	0.613
DBP (mmHg)	78 ± 4	78 ± 2	0.374	0	81 ± 4	77 ± 3	0.268	4.9	0.665
MAP (mmHg)	89 ± 4	89 ± 2	0.998	0	90 ± 3	88 ± 3	0.559	2.2	0.675
SV (mL)	65 ± 5	67 ± 6	0.595	3.1	70 ± 5	70 ± 6	0.963	0	0.66
CO (L/min)	3.9 ± 0.39	4.3 ± 0.34	0.217	10.2	4.5 ± 0.3	4.4 ± 0.3	0.719	2.3	0.383
TPR	1699 ± 115	1706 ± 112	0.945	0.4	1635 ± 123	1627 ± 113	0.895	0.5	0.76

Δ: % change from baseline; BMI: body mass index; HbA_1c_: glycated haemoglobin; HR: heart rate; SBP: systolic blood pressure; DBP: diastolic blood pressure; MAP: mean arterial pressure; SV: stroke volume; CO: cardiac output; TPR: total peripheral resistance.

* *p* < 0.05 significance.

## Relationship between glycaemic control and cardiovascular autonomic function

**Figure 2.**
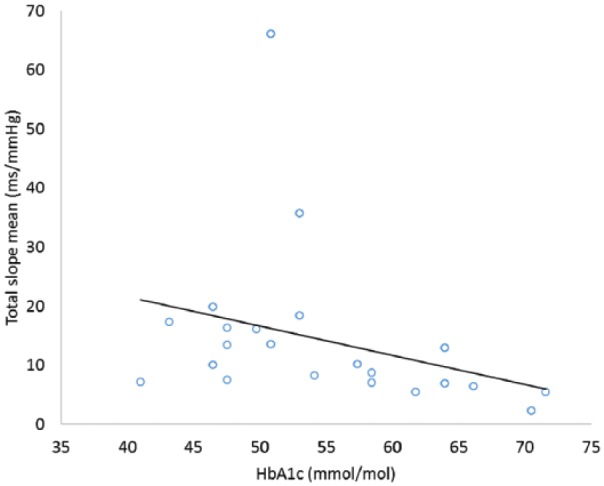
Relationship between glycaemic control and BRS at baseline. BRS: baroreflex receptor sensitivity.

There was a significant negative association between HbA_1c_ and BRS (*r_s_* = –0.592, *p* = 0.004; Figure 2). However, there was no significant relationship between HbA_1c_ and other measures of autonomic regulation.

## Effects of HIIT on cardiovascular autonomic function

HIIT was associated with a 21% decrease in the HF component of systolic BPV (*p* = 0.003; Table 3). There was no significant difference between groups in measures of HRV, BPV, RRI and SDNN, other than a worsening of SBP HF% (HIIT: 19 ± 4 to 15 ± 3 vs Control: 28 ± 5 to 28 ± 3, >0.01; Table 3). There were >10% changes to RRI LF/HF, SBP LF/HF and DBP LF/HF, but these changes were observed in both the HIIT and control groups.

**Table 3.** The effect of HIIT on cardiovascular autonomic measures.

	Control (*n* = 11)				Intervention (*n* = 11)				Between-group *p* value
	Baseline	Post-treatment	*p*-value	Δ (%)	Baseline	Post-treatment	*p*-value	Δ (%)	
Heart rate variability									
RRI (ms)	920 ± 6	930 ± 32	0.505	1.1	954 ± 49	973 ± 53	0.386	2	0.672
SDNN (ms)	42 ± 3	56 ± 14	0.424	33	53 ± 14	53 ± 15	0.962	0	0.340
RRI LFnu (%)	53 ± 6	51 ± 5	0.509	3.8	47 ± 6	48 ± 5	0.477	2.1	0.992
RRI HFnu (%)	47 ± 6	49 ± 5	0.549	4.3	53 ± 6	52 ± 5	0.477	1.9	0.984
RRI LF/HF	1.20 ± 0.29	1.0 ± 0.17	0.11	16.7	0.9 ± 0.21	0.73 ± 0.07	0.308	18.9	0.203
Blood pressure variability									
SBP LFnu (%)	37 ± 3	36 ± 2	0.755	2.7	34 ± 3	33 ± 2	0.681	2.9	0.608
SBP HFnu (%)	28 ± 5	28 ± 3	0.954	0	19 ± 4	15 ± 3	0.021*	21	0.003**
SBP LF/HF	1.06 ± 0.26	0.91 ± 0.14	0.281	14.2	0.86 ± 0.21	0.73 ± 0.1	0.328	15.1	0.169
DBP LFnu (%)	41 ± 3	39 ± 2	0.456	4.9	35 ± 4	34 ± 3	0.53	2.9	0.24
DBP HFnu (%)	21 ± 5	21 ± 4	0.892	0	21 ± 6	20 ± 6	0.894	4.8	0.884
DBP LF/HF	1.19 ± 0.29	1.01 ± 0.17	0.139	15.1	0.91 ± 0.21	0.73 ± 0.07	0.286	19.8	0.200
Baroreflex receptor sensitivity									
Total slope mean (ms/mmHg)	10.8 ± 1.4	10.3 ± 1.0	0.716	4.6	18.0 ± 5.5	19.1 ± 4.75	0.093	6.1	0.150

Δ: % change from baseline; RRI: R-R interval; SDNN: standard deviation of R-R interval; LFnu: low-frequency normalised units; HFnu: high-frequency normalised units; LF/HF: low frequency:high frequency ratio; SBP: systolic blood pressure; DBP: diastolic blood pressure.

* *p*<0.05 significance,^**^ *p* < 0.01 significance.

## Discussion

This is the first RCT designed to investigate the impact of HIIT on cardiovascular autonomic function in adults with type 2 diabetes. The main findings in this study suggest that (1) poor glycaemic control was associated with decreased BRS and (2) HIIT improved HbA_1c_ levels, but had no effect on measures of cardiovascular autonomic function. These findings suggest that while poor glycaemic control was associated with decreased BRS, short-term improvements in glycaemic control achieved through HIIT were not associated with improvements in cardiovascular autonomic function.

Previous research has demonstrated a relationship between hyperglycaemia and cardiovascular autonomic neuropathy; however, the direction of causation is unclear. Increased sympathetic activity may precede insulin resistance,^25^ whereas oxidative stress caused by hyperglycaemia has been linked to neuronal dysfunction and ischaemia.^26^ Also impairment of nitric oxide (NO) due to hyperglycaemia may lead to reduction of neurovascular perfusion and ultimately apoptosis.^26^ It is likely that improvements in glycaemic control could benefit cardiovascular autonomic neuropathy in the long term, and lifestyle interventions to reduce hyperglycaemia in type 2 diabetes patients are recommended before any pharmacological intervention.^6^

Twelve weeks of HIIT in type 2 diabetes patients improved HbA_1c_, but there was no improvement in cardiovascular autonomic function. This was consistent across time and frequency-domain measures of HRV and BPV. To our knowledge, there have been two previous studies investigating HRV following HIIT in type 2 diabetes patients.^27,28^ An improvement in RRI and its standard deviation was observed following 12 weeks of HIIT, but there was no control group.^27^ In addition, 16 weeks of supervised HIIT improved both HF and LF HRV, and this seemed to be larger when compared to moderate-intensity continuous exercise.^28^ Although detailed mechanisms behind these changes have not been confirmed, the following have been suggested. HIIT is a potent stimulator of NO bioavailability due to the large shear stress experienced in the vessels,^28^ and NO is a modulator of cardiac vagal activity. In addition, increases in blood volume following training can increase cardiac vagal modulation, via baroreflex activation.^29^ These may occur to a greater extent following HIIT compared to moderate-intensity continuous training.

In contrast to the aforementioned studies, no significant changes in measures of cardiovascular autonomic function were observed in this study. This may be due to numerous factors. A systematic review on the effect of exercise training on HRV in patients with type 2 diabetes revealed that short-term interventions (3–4 months) had less effect than exercise programmes of longer duration.^9^ Hottenrott et al.^30^ concluded that exercise programmes longer than 3 months are needed to stimulate changes to vagal modulation in both healthy and disease populations. In another review of exercise studies on cardiovascular autonomic function in type 2 diabetes,^8^ the majority of exercise studies (of which none adopted HIIT) demonstrated improvements in autonomic function following exercise; however, only 4 out of 18 studies were RCTs, suggesting that these findings should be considered with caution due to limitations in study design. Three of the studies found no improvement in autonomic function following exercise. In one of those studies, 16 weeks of aerobic training improved HRV in obese individuals, but not in obese individuals with type 2 diabetes. This may suggest limited capacity and potential plasticity of the autonomic nervous system to respond to exercise training in type 2 diabetes.^30^ This study was designed to measure the impact of HIIT rather than weight loss on glycaemic control. The intervention group had to therefore increase their calorie intake to maintain weight during the 12 weeks. Between-group analysis revealed no significant reduction in weight following HIIT, and it has previously been reported that weight loss is related to improvements in cardiovascular autonomic function. Indeed, it has been shown that a minimal exercise intensity of 8 kcal kg per week is needed for changes in HRV.^30^

It has previously been shown in type 2 diabetes that prolonged (i.e. 52 weeks) combined aerobic and resistance exercise reduced HbA_1c_, which correlated with improvements in BRS.^31^ Changes in central haemodynamics did not correlate with improvements in BRS, indicating that improved glucose control drives improvements in cardiovascular autonomic function more than central hemodynamics.^31^ In this study, the significant between-group improvement in HbA_1c_ cannot solely be attributed to an improvement in the intervention group, but also a decline in glycaemic control in the non-exercise group. This may explain the lack of change in cardiovascular autonomic function following HIIT.

Several limitations of the study need to be noted. The study was powered to detect a change in HbA1c and therefore could have been underpowered to identify changes in autonomic function. That being said, significant changes in HRV have been previously identified with similar sample sizes.^27^ A longer intervention designed to target weight loss may be preferable for both metabolic and cardiovascular improvements. Clinically, meaningful changes in a number of HRV and BPV measures were detected in both the control and intervention groups. It is not certain why the control group showed such differences, and although the sample size was sufficient to detect a significant change in HbA_1c_ in response to HIIT, it appears that a larger sample size will be needed to detect significant changes between groups considering the large standard deviations of these measures.

## Conclusion

In conclusion, hyperglycaemia is related to worse cardiovascular autonomic function in individuals with type 2 diabetes. Unsupervised HIIT over 12 weeks may improve glycaemic control but has limited effect on measures of cardiovascular autonomic regulation including HRV and BPV. Future research is warranted to address the effect of long-term HIIT on cardiovascular autonomic function in individuals living with type 2 diabetes.
